# Alone in a Crowd: Is Social Contact Associated with Less Psychological Pain of Loneliness in Everyday Life?

**DOI:** 10.1007/s10902-023-00661-3

**Published:** 2023-05-04

**Authors:** Olga Stavrova, Dongning Ren

**Affiliations:** 1 Tilburg University, Warandelaan 2, 5000 LE, Tilburg, Netherlands; 2 Emory University, Atlanta, USA

**Keywords:** Loneliness, Well-being, Social contact, Social withdrawal, Experience sampling, Social interaction quality

## Abstract

People are often advised to engage in social contact to cope with the experience of loneliness and improve well-being. But are the moments of loneliness actually more bearable when spent in other people’s company? In this research, we proposed and tested two conflicting theoretical accounts regarding the role of social contact: social contact is associated with a stronger (the amplifying account) or with a weaker (the buffering account) negative effect of loneliness on psychological well-being. Analyses of three datasets collected using ecological momentary assessments (*N*_individuals_ = 3,035) revealed that the negative association between loneliness and well-being was stronger when participants were with others than alone, consistent with the amplifying account. Further, when participants experienced high levels of loneliness, being with others was associated with the same or with even a lower level of well-being than being alone. These findings suggest that simply spending time with others (vs. alone) is not associated with a reduced burden of loneliness and may even backfire.

**Supplementary Information:**

The online version contains supplementary material available at 10.1007/s10902-023-00661-3.

Loneliness has been associated with poor health and psychological well-being, including a lower life satisfaction, self-esteem and meaning in life, and higher depression rates (Cacioppo et al., 2002, 2010; Çivitci & Çivitci, 2009; Goodwin et al., 2001; Qualter et al., 2010; Stillman et al., 2009; VanderWeele et al., 2012). Feeling lonely throughout the day is associated with lower positive affect, higher anxiety and psychological distress (Hawkley et al., 2003; Yung et al., 2021).

The defining feature of loneliness, which is often considered responsible for the negative consequences that loneliness brings about, is the discrepancy between the desired and actual social relationships (Russell et al., 1980). Consequently, to tackle loneliness and increase psychological well-being, one common advice that self-help books and popular press offer is to improve one’s social engagement such as joining a group or establishing new connections (DCMS, 2021). However are the moments of loneliness actually more bearable when spent in others’ company than alone? Surprisingly, past work has not examined this question directly. In the present research, building on the existing theoretical and empirical work on loneliness and well-being, we propose two possible, opposing predictions regarding the role of social contact: social contact may be associated with a weaker (the buffering account) or a stronger (the amplifying account) negative effect of loneliness on psychological well-being. We put these two theoretical accounts to test using three large datasets of ecological momentary assessments.

## Buffering Account: The Negative Links Between Loneliness and Well-Being are Weaker when in Others’ Company

Social relationships are one of the most important predictors of psychological well-being (Diener & Ryan, 2009). Social relationships are an important source of life satisfaction and meaning in life (Lucas & Dyrenforth, 2006; Stavrova & Luhmann, 2016; Sun et al., 2020), frequent (vs. rare) social contact predicts higher psychological well-being (Ren et al., 2021a, b), better physical health and longevity (Stavrova & Ren, 2020), whereas social isolation is associated with psychological distress and mental illness (Marinucci & Riva, 2021). Besides having a direct positive effect on well-being, social contact may serve as a protective factor of individuals’ well-being against negative stressors (Cohen & Wills, 1985). For example, in the context of workplace, positive social relationships mitigate the negative consequences of occupational stress (Kinman et al., 2011; Kirmeyer & Dougherty, 1988; LaRocco et al., 1980; Pluut et al., 2018; Viswesvaran et al., 1999). In an experience sampling study, adolescents reported less loneliness when entering their friends’ (but not their family’s) company after being alone (relief effect; van Roekel et al., 2015). The buffering effect of social relationships has also been documented in studies of stressful and traumatic life events. For example, social connectedness helps individuals cope with bereavement (Vanderwerker & Prigerson, 2004), social support protects against the negative consequences of acculturative stress (Choi, 1997), and friend support on social media alleviates the psychological impact of stressful life events (Zhang, 2017).

In line with these findings, social contact may serve a similar buffering function when people feel lonely—a painful and distressing experience (Rokach, 1988). Several studies provided initial support to this possibility. For example, in work context, a high level of organizational support represents a protective factor that prevents employee loneliness from damaging their psychological well-being (Mohapatra et al., 2020). In older individuals, continued employment (compared to retirement), which represents a source of social contact in older age, reduces the negative impact of loneliness on depressive symptoms (Segel-Karpas et al., 2018). Drawing from these findings, social contact could mitigate the negative effect of momentary loneliness on psychological well-being in everyday life as well.

## Amplifying Account: The Negative Links Between Loneliness and Well-Being are Stronger when in Others’ Company

In contrast to the buffering account, the opposite prediction can be generated based on the literature on social consequences of loneliness (Cacioppo & Hawkley, 2005). That is, loneliness might feel especially aversive when being in others’ company (vs. alone). We refer to this prediction as the amplifying model of social contact. There are two potential ways through which social contact could be linked to a particularly painful psychological experience of loneliness: Unfulfilled social withdrawal desire and Decreased interaction quality.

### Unfulfilled Social Withdrawal Desire

Loneliness contributes to the perception of the social world and the behavior of others as potential threat (Hawkley et al., 2007; Spithoven et al., 2017). For example, brief episodes of social exclusion (an established cause of loneliness; Leary 1990) increase people’s desire to be alone (Ren et al., 2016, 2021a, b). Chronic (or dispositional) loneliness is associated with lower levels of enthusiasm for social interactions (Vanhalst et al., 2015) and greater social withdrawal tendencies (Nurmi et al., 1996; Watson & Nesdale, 2012). Hence, when feeling lonely, people might experience a greater preference for being alone; and the presence of others or having to engage in social interactions under these circumstances might feel particularly burdensome and aggravate the unpleasant feeling of loneliness. Therefore, engaging in social contact while feeling lonely could go hand in hand with lower psychological well-being.

### Decreased Interaction Quality

Loneliness might undermine the psychological rewards of social interactions by negatively affecting the interaction quality. Loneliness predisposes people to approach social interactions with cynicism, distrust, and an expectation of rejection and betrayal (Rotenberg 1994). Such expectations might negatively shape lonely people’s behavior towards others. For example, lonely people devote less attention to their interaction partner and approach social situations with caution and anxiety (Knowles et al., 2015; Lucas et al., 2010). Maladaptive social behaviors associated with loneliness might in turn negatively affect other people’s behavior towards them (Cacioppo & Patrick, 2008). Indeed, multiple studies have documented a social stigma of loneliness. People see loneliness as a signal of a target’s inability to establish social connections, which could be indicative of some personal flaws and induce ostracism (Cacioppo & Hawkley, 2005). Several studies showed that lonely people are perceived more negatively by others (Lau & Gruen, 1992; but see Kerr & Stanley 2021a, b), are attributed a lower psychosocial adjustment and are more likely to be ostracized (Rotenberg & Kmill, 1992). Analyses of dyadic interactions showed that people who were told that their interaction partner was lonely, ascribed their partner a lower sociability and were less social towards them themselves (Rotenberg et al., 2002). Studies of social networks showed that this effect emerged also when individuals were not explicitly told about others’ loneliness: individuals report more negative perception of their new acquaintances who self-report higher (vs. lower) loneliness (Jones et al., 1983; Tsai & Reis, 2009). It is possible that maladaptive interpersonal behaviors associated with loneliness (and not loneliness per se) elicit negative behavioral reactions from others (Kerr & Stanley, 2021a, b). Taken together, these arguments suggest that individuals who engage in social contact in moments of loneliness might have poor quality social interactions, which would undermine their psychological well-being.

## Daily Life Approach and Ecological Validity

In the present research, we tested the two conflicting theoretical accounts of the role of social contact in the relationship between loneliness and well-being: the buffering account and the amplifying account. We adopted “the everyday life” approach by using ecological momentary assessments: participants provided information about several randomly selected episodes during their day, including their experience of loneliness, psychological well-being and the presence of others during each episode. This approach allowed us to test whether the negative association between the experience of loneliness and psychological well-being is attenuated or strengthened when others are present (vs. alone) during each episode. This approach has several advantages: it captures people’s moment-to-moment experiences in a naturalistic setting enhancing external validity, it minimizes memory and recall biases by using a real-time assessment, and it allows for studying within-person processes by recording multiple observations from each participant on several occasions (Santangelo et al., 2013).

Having acknowledged these strengths, it is important to be aware that this approach does not provide causal evidence (we return to this point in the General Discussion). Despite this shortcoming, it allows us to fulfil the present research goal: by observing the associations between the experience of loneliness and psychological well-being as they occur in daily life, we can determine whether these associations are weaker in others’ presence (and therefore consistent with the buffering account) vs. stronger in others’ presence (and therefore consistent with the amplifying account). Hence, using ecological momentary assessments will allow us to determine whether the moments of loneliness feel better or worse when spent in others’ company (vs. alone).

## The Present Research

We analyzed three large datasets of ecological momentary assessments (*N*_total individuals_ = 3,035). Study 1 used a large nationally representative sample of German adults who provided a detailed account of their daily experiences using Day Reconstruction Method (DRM). Study 2 replicated the findings in a seven-day long Experience Sampling Method (ESM) study. Study 3 used another ESM dataset that has been recently collected during the COVID-19 pandemic. The studies were not pre-registered. We report how we determined our sample size, all data exclusions (if any), all manipulations, and all measures in the studies. All analyses scripts, all study materials, and the data of Study 3 can be accessed at OSF (https://osf.io/gfe7k/?view_only=4dafec27aded48ad82e3935b71bb05cb). The data of Studies 1 and 2 can be accessed at DIW Berlin: https://www.diw.de/en/diw_01.c.678568.en/research_data_center_soep.html.

## Study 1

### Method

#### Participants

 We used the data from the German Socio-Economic Panel - Innovation Sample (GSOEP-IS) (Richter & Schupp, 2015). The Innovation Sample is a sub-sample of the larger nationally representative annual panel study (German Socio-Economic Panel) that has been conducted since 1984 and includes more than 20,000 individuals (Wagner et al., 2007). The Innovation Sample consists of ~ 2,000 individuals and is also representative of the German population aged 16 and higher. It is used to examine innovative research questions using novel methods, such as DRM. 2,498 individuals (*M*_age in 2012_ = 49.78, *SD*_age_ = 19.10, 47.3% male) completed the DRM module annually between 2012 and 2015. Every time, participants provided information about three daily episodes, resulting in the overall number of observations *N* = 21,652. A sensitivity analysis showed that this sample size provides a 98% power to detect a significant (α = 0.05) interaction between momentary loneliness and social contact (unstandardized *b* = − 0.05; calculated with nlme package; Galecki & Burzykowski 2013). Participants were compensated with 10€ (DIW Berlin, 2014).

#### Procedure and Measures

Participants were asked to reconstruct their previous day by breaking it into episodes and answering a range of questions about each episode. Participants indicated their location, whether they were alone or in social company. For each participant, three randomly selected episodes were chosen for a detailed assessment that included measures of well-being and loneliness.

For each episode, participants indicated whether someone else was present (1 = with others, 0 = alone)^1^. To measure loneliness, participants indicated how lonely they felt during each episode (1 = not at all, 7 = very strong). Using the same 7-point scale, participants reported the intensity of their feelings during the episode by rating the following items: happiness, satisfaction, enthusiasm, meaning, anger, sadness, worries, stress, boredom, pain, fatigue, frustration. We averaged participants’ responses to these items to form a composite score of psychological well-being (responses to negative items were reverse-coded; Cronbach’s α = 0.74)^2^.

### Results

Descriptive statistics and zero-order correlations among the variables are shown in Table 1.

**Table 1:** Means, standard deviations, and correlations

	Study 1				Study 2			
Variable	*M*	*SD*	1	2	*M*	*SD*	1	2
1. Others’ presence	0.45	0.50	-	-	0.66	0.47	-	-
2. Momentary loneliness	1.40	1.02	− 0.13***	-	1.46	1.12	− 0.18***	-
			[-0.14, − 0.12]				[-0.19, − 0.16]	
3. Momentary well-being	5.40	0.69	0.13***	− 0.36***	5.27	0.80	0.10***	− 0.41***
			[0.12, 0.15]	[-0.37, − 0.35]			[0.08, 0.12]	[-0.42, − 0.39]

*Note.* ***p < .001. Others’ presence: 1 = with others, 0 = alone; Numbers in the brackets are 95% confidence intervals.

**Fig. 1:**
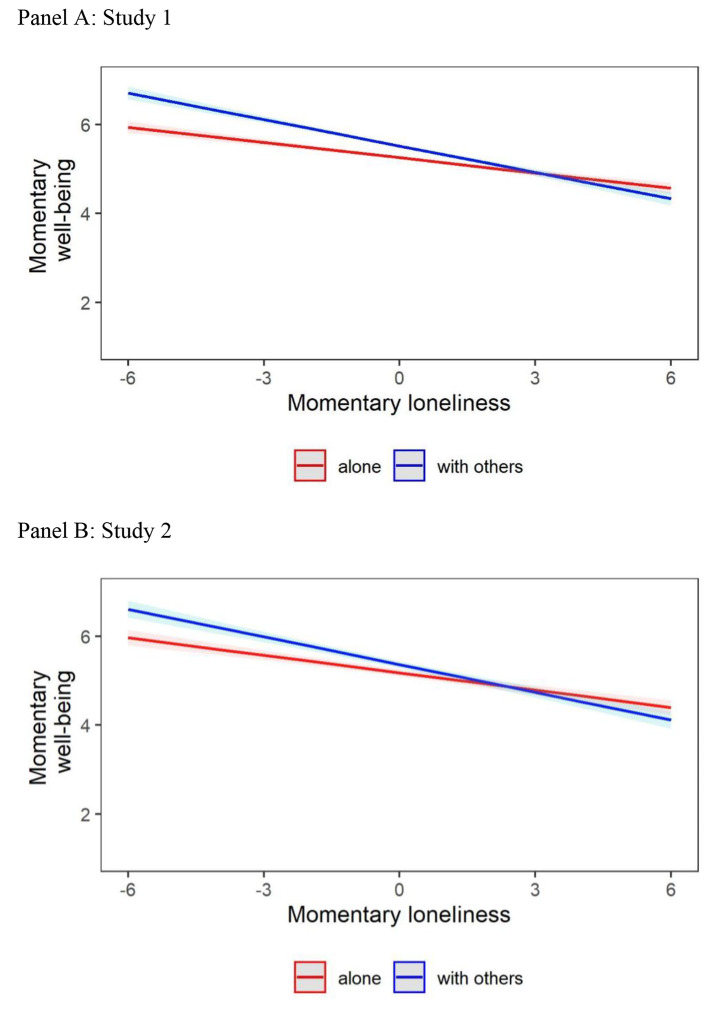
Momentary loneliness, momentary social contact and momentary psychological well-being *Note*. Momentary loneliness and momentary social contact were centered within participants.

For the main analyses, we used multilevel regression with momentary loneliness, the presence of others, and their interaction term as predictors, and momentary well-being as the outcome; random intercepts were estimated at the level of participants and years. Following recent recommendations in the methodological literature (Yaremych et al., 2021), we used group-mean (that is, person-level) centering for all predictors. We report unstandardized regression coefficients (*b*) that reflect within-individual changes. We specified an error structure that allowed for correlations between adjacent time points for the same participant (Finch et al., 2019) using the nlme package in R (Pinheiro et al., 2021). Model results are shown in Table 2. The interaction between momentary loneliness and momentary social contact reached significance (*b* = -0.05, *p* < .001). The interaction is plotted on Fig. 1. A simple slope analysis showed that the negative association between momentary loneliness and momentary well-being was stronger in others’ presence (*b* = -0.20, *p* < .001) than alone (*b* = -0.13, *p* < .001), providing support to the amplifying account. We further tested the effect of social contact (vs. alone) at low and high levels of loneliness. As expected, when people experienced low levels of loneliness (the lowest empirical loneliness score), they reported higher well-being when they were in others’ company than when they were alone (*b* = 0.33, *p* < .001). Interestingly, when people experienced high levels of loneliness (the highest empirical loneliness score), the positive association between social contact and well-being became negative and nonsignificant (*b* = -0.11, *p* = .10), suggesting that people no longer felt better in others’ company than alone during moments of loneliness. Finally, the interaction between momentary loneliness and momentary social contact remained robust against controlling for gender and age (Table S3 in the Supplementary materials).

**Table 2:** Multilevel regression predicting momentary psychological well-being

	Study 1			Study 2		
*Predictors*	*b*	*95% CI*	*p*	*b*	*95% CI*	*p*
Momentary loneliness	-0.16	[-0.17, -0.15]	**< 0.001**	-0.17	[-0.18, -0.16]	**< 0.001**
Others’ presence	0.14	[0.13, 0.16]	**< 0.001**	0.09	[0.07, 0.11]	**< 0.001**
Momentary loneliness x Others’ presence	-0.05	[-0.07, -0.02]	**< 0.001**	-0.04	[-0.06, -0.01]	**0.003**
N years	4			-		
N individuals	2,498			265		
N assessments	21,652			12,730		

*Note.* Others’ presence: 1 = with others, 0 = alone. All predictors were group-mean centered (i.e., within individuals).

### Discussion

Study 1 provided support to the amplifying account: the negative association between loneliness and well-being was stronger in others’ presence than alone. Interestingly, the results also showed that even though being around others (relative to being alone) was associated with higher well-being on average, this beneficial effect of sociality disappeared at high levels of loneliness. These findings suggested that when individuals felt lonely, the presence (vs. absence) of others was no longer associated with higher well-being.

## Study 2

The goal of Study 2 was to replicate the findings of Study 1 using a slightly different method: Experience Sampling Method.

### Method

#### Participants

In 2014, members of the GSOEP-IS were invited to participate in the Experience Sampling Module (ESM). In the ESM module, participants were asked to complete seven brief surveys at random times every day, for the period of 7 days. Participants were compensated with 1€ for each completed survey and an additional 4€ for each day when they completed all seven daily surveys (Bohlender & Glemser, 2017). 265 individuals (*M*_age in 2014_ = 47.80, *SD*_age_ = 17.83, 43.8% male) participated, providing *N* = 12,730 observations. A sensitivity analysis (same as in Study 1) showed that this sample size provides an 85% power (momentary loneliness x social contact interaction; unstandardized *b* = − 0.04).

#### Procedure and Measures

Every day participants were sent random notifications on their phone asking them to complete a brief survey about their momentary experiences. Every time, participants were asked to indicate whether they were currently alone or with others (1 = with others, 0 = alone), how lonely they felt in the moment (1 = not at all, 7 = very strong) and to what extent they felt different emotions (1 = not at all, 7 = very strongly; the same items were used as in Study 1). Following the procedure of Study 1, we reverse-coded negative items and averaged all emotion items to measure psychological well-being (Cronbach’s α = 0.82).

### Results

Descriptive statistics and zero-order correlations among the variables are shown in Table 1.

We used the same analytic strategy as in Study 1. Model results are shown in Table 2. Like in Study 1, there was a significant interaction between momentary loneliness and the presence of others (*b* = -0.04, *p* = .003). The interaction is plotted in Fig. 1 (Panel B). The negative association between momentary loneliness and momentary well-being was stronger in others’ presence (*b* = -0.21, *p* < .001) than alone (*b* = -0.13, *p* < .001). In addition, at low levels of loneliness (the lowest empirical loneliness score), being in others’ company was associated with higher well-being than being alone (*b* = 0.28, *p* < .001); in contrast, at high levels of loneliness (the highest empirical loneliness score), others’ presence was no longer associated with better well-being (*b* = -0.13, *p* = .078). Like in Study 1, adding gender and age to the model did not change this pattern (Table S3 in the Supplementary materials).

### Discussion

Study 2 replicated the findings of Study 1 using an experience sampling method. Consistent with the amplifying account, momentary loneliness was more strongly associated with worse well-being when others were present than alone. Additionally, the association between others’ presence and well-being was no longer positive in moments of loneliness, suggesting that when people felt lonely, being around others no longer felt better than being alone.

## Study 3

Study 3 aimed to replicate the amplifying effect of social interactions using a novel ESM sample we recently collected during the COVID-19 pandemic. Increased loneliness and mental health problems are among the most often mentioned consequences of the global health crisis (Brooks et al., 2020), rendering the question of the role of social contact in the links between loneliness and well-being particularly relevant in the pandemic context.

Here, we also explored the role of social withdrawal desire and interaction quality. First, we tested whether feeling lonely is associated with an increased desire to be alone which could feel particularly aversive when in other people’s company (vs. alone). Second, we explored whether when feeling lonely, people tend to have social interactions of poorer quality, and whether poor interaction quality is associated with worse well-being. Note that given the correlational nature of these data, the goal of these analyses was not to test the causal relationships outlined above bur rather to evaluate whether the pattern of correlations in the data is consistent with these theorized causal relationships or not. For example, a lack of the correlation between loneliness and social interaction quality would be inconsistent with the assumption that when feeling lonely, people tend to have social interactions of poorer quality and would therefore make this causal model unlikely.

### Method

#### Participants

For this study, we recruited 454 UK residents on Prolific Academic in August 2020. 146 participants failed an attention check question (see SOM) and were dismissed, while the remaining 308 participants were invited to take part in the 7-day long ESM study. 272 participants completed at least one ESM assessment and constituted the final sample (*M*_age_ = 34.33, *SD*_age_=12.47, 25% male). Overall, 272 individuals contributed 7,933 assessments. A sensitivity analysis (same as in Studies 1 and 2) showed that this sample size provides a 99% power (momentary loneliness x social contact interaction; unstandardized *b* = − 0.11). Participants earned 1.5 pounds per day if they completed at least five daily surveys; participants who completed all daily surveys were paid an additional bonus of 3 pounds.

#### Procedure and Measures

Participants downloaded a smartphone application (ethicadata.com) that was programmed to send time-triggered push notifications asking them complete 5-minute surveys on their phone. Participants were sent five notifications daily for a period of seven days, resulting in 35 momentary assessments overall. The notifications were sent randomly within the following time intervals: 9:20 − 11:40 (first assessment), 11:40 − 14:00 (second assessment), 14:00–16:20 (third assessment), 16:20 − 18:40 (fourth assessment), 18:40 − 21:00 (fifth assessment). Participants completed 31.09 (*SD* = 5.61) assessments on average (with an average of 4.59 [*SD* = 0.83] assessments per day).

All momentary measures asked about participants’ experiences within the last hour and were administered in a random order.

Participants indicated whether they were interacting with others during the last hour (1 = yes, 0 = no). To measure *momentary loneliness*, participants indicated to what extent they felt lonely during the last hour (1 = not at all, 5 = a great deal). To measure *psychological well-being*, participants indicated to what extent they felt happy, satisfied with life, that their life was meaningful, angry, sad, and bored during the last hour (1 = not at all, 5 = a great deal). After reverse-coding the negative items, we averaged all 6 items into a composite measure of psychological well-being (Cronbach’s α = 0.79).

To measure *momentary social withdrawal desire*, participants completed two items (“During the last hour, to what extent have you craved more time alone?” and “During the last hour, did you want to be alone?”; 1 = not at all, 5 = a great deal). These items were averaged to form a composite (*r* = .66, *p* < .001). To measure *quality of social interactions*, participants also indicated whether “they had negative interactions with others, during the last hour” (1 = not at all, 5 = a great deal)^3^.

### Results

Descriptive statistics and correlations among variables are presented in Table 3.

**Table 3:** Means, standard deviations, and correlations, Study 3

Variable	*M*	*SD*	1	2	3	4
1. Others’ presence	0.78	0.42	-	-	-	-
2. Momentary loneliness	1.30	0.67	− 0.10***	-	-	-
			[-0.13, − 0.08]			
3. Momentary well-being	3.63	0.67	0.14***	− 0.48***	-	-
			[0.11, 0.16]	[-0.49, − 0.46]		
4. Momentary social withdrawal desire	1.49	0.81	− 0.08***	0.28***	− 0.40***	-
			[-0.10, − 0.06]	[0.26, 0.30]	[-0.42, − 0.38]	
5. Momentary negative interactions	1.21	0.58	0.10***	0.26***	− 0.31***	0.32***
			[0.08, 0.12]	[0.24, 0.28]	[-0.33, − 0.29]	[0.30, 0.34]

*Note.* ***p < .001. Others’ presence: 1 = with others, 0 = alone. Numbers in the brackets are 95% confidence intervals.

**Fig. 2:**
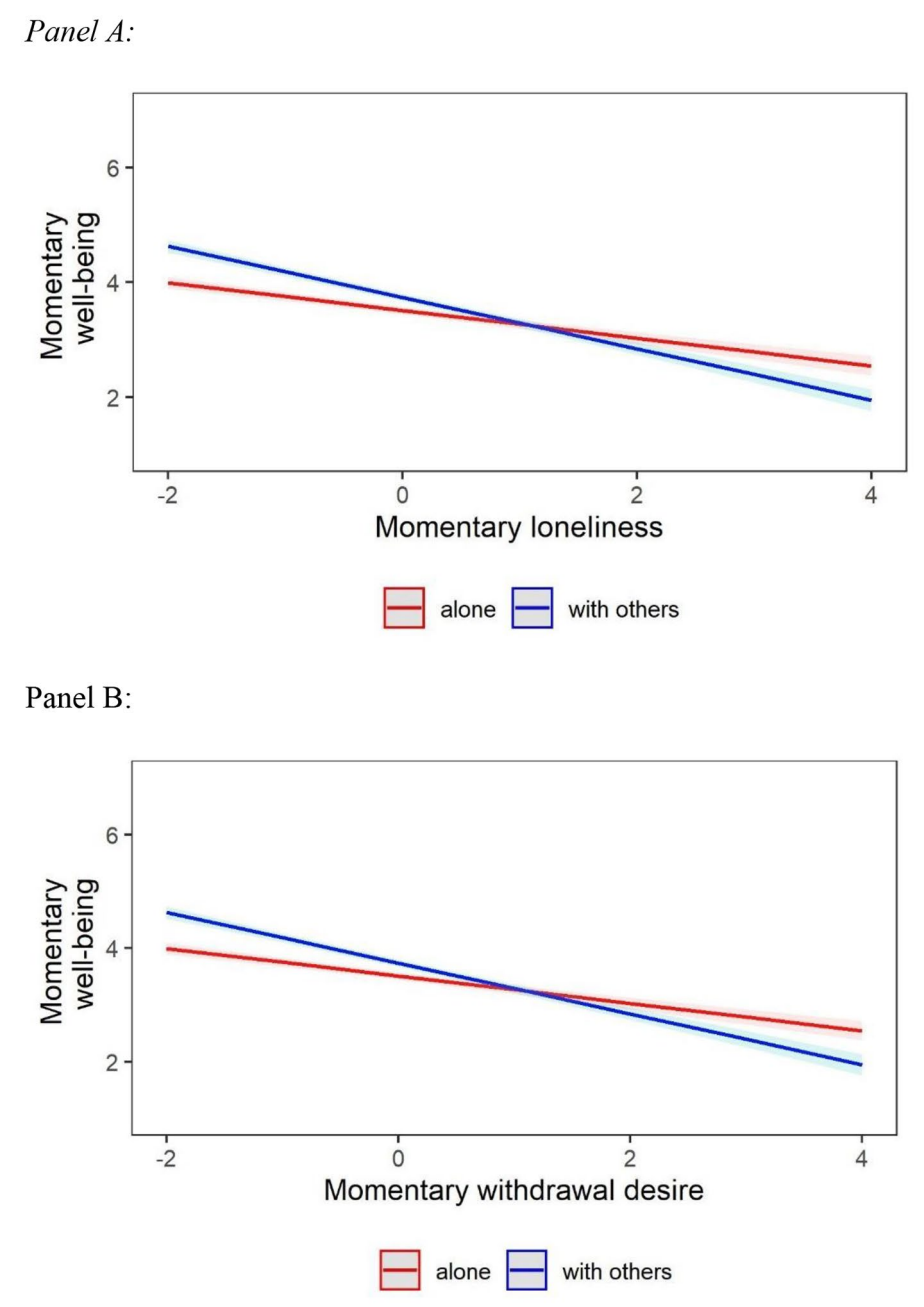
Momentary loneliness (**Panel A**), momentary social withdrawal desire (**Panel B**), momentary social contact and momentary well-being, Study 3 *Note*. Momentary loneliness, momentary social withdrawal desire and momentary social contact were centered within participants.

First, we sought to replicate the moderating role of social contact on the link between momentary loneliness and well-being. We used the same analytic strategy as in the first two studies. The results are shown in Table 4 (Model 1) and Fig. 2 (Panel A). The interaction between momentary loneliness and momentary social interactions was significant (*b* = -0.11, *p* < .001). Consistent with the amplifying account, the negative association between momentary loneliness and momentary well-being was stronger in the presence of others (*b* = -0.45, *p* < .001) than alone (*b* = -0.24, *p* < .001). In addition, at low levels of loneliness (the lowest empirical loneliness score), being around others was associated with higher well-being than being alone (*b* = 0.33, *p* < .001); in contrast, at high levels of loneliness (the highest empirical loneliness score), being around others was associated with worse well-being than being alone (*b* = -0.28, *p* < .001). In further analyses, we added gender, age and employment status as additional control variables, which did not change the focal interaction effect (see Table S4 in the Supplementary materials).

**Table 4:** *Multilevel regression results, Study 3*

	Model 1			Model 2			Model 3			Model 4			Model 5		
	Momentary well-being			Momentary withdrawal desire			Momentary well-being			Momentary negative interactions			Momentary well-being		
*Predictors*	*b*	*95% CI*	*p*	*b*	*95% CI*	*p*	*b*	*95% CI*	*p*	*b*	*95% CI*	*p*	*b*	*95% CI*	*p*
Momentary loneliness	-0.34	-0.36 – -0.33	**< 0.001**	0.31	0.29–0.34	**< 0.001**				0.20	0.18–0.22	**< 0.001**	-	-	-
Others’ presence	0.12	0.09–0.14	**< 0.001**	-	-	-	0.13	0.10–0.15	**< 0.001**	-	-	-	-	-	-
Momentary loneliness x Others’ presence	-0.11	-0.15 – -0.07	**< 0.001**	-	-	-				-	-	-	-	-	-
Momentary withdrawal desire	-	-	-	-	-	-	-0.27	-0.28 – -0.25	**< 0.001**	-	-	-	-	-	-
Momentary withdrawal desire x Others’ presence	-	-	-	-	-	-	-0.20	-0.24 – -0.16	**< 0.001**	-	-	-	-	-	-
Momentary negative interactions	-	-	-	-	-	-	-	-	-	-	-	-	-0.31	-0.32 – -0.29	**< 0.001**
N individuals	272			272			272			272			272		
N assessments	7933			7580			7579			7937			7932		

*Note.* Others’ presence: 1 = with others, 0 = alone. All predictors were group-mean centered (i.e., within individuals). Model 1 tests whether the effect of momentary loneliness is stronger in others’ presence (vs. alone). Model 2 tests whether momentary loneliness predicts a stronger social withdrawal desire and Model 3 tests whether a stronger momentary withdrawal desire predicts lower well-being and whether this effects is stronger in others’ presence (vs. alone). Model 4 tests whether momentary loneliness predicts more negative social interactions and Model 5 tests whether more negative social interactions predict lower well-being

Next, we estimated a series of multilevel regression models to explore the relationships between momentary loneliness, momentary social withdrawal desire, momentary negative interactions and others’ presence (vs. being alone). These results are shown in Table 4.

#### Social Withdrawal Desire

When feeling lonely, people might experience a greater preference for being alone; the presence of others in such moments might feel particularly burdensome and aggravate the unpleasant feeling of loneliness. Consistent with this possibility, momentary loneliness was positively associated with momentary withdrawal desire, *b* = 0.31, *p* < .001 (Table 4, Model 2). The stronger the withdrawal desire, the lower was the experienced well-being (*b* = -0.27, *p* < .001), in particular in others’ presence (interaction between others’ presence and withdrawal desire: *b* = -0.20, *p* < .001), see Table 4 (Model 3). When participants wanted to be with others (i.e., were at their lowest social withdrawal desire), they reported higher well-being when being around others vs. alone (*b* = 0.72, *p* < .001); when participants wanted to be alone (i.e., were at their highest social withdrawal desire), they reported higher well-being when being alone than with others (*b* = -0.59, *p* < .001); see Fig. 2, Panel B. These results are the same when controlling for basic socio-demographics (age, gender, employment status). See Table S4 in the Supplementary materials.

#### Quality of Social Interactions

Table 4 further demonstrates that the lonelier people felt in the moment, the more likely they were to have negative social interactions (Model 4: *b* = 0.20, *p* < .001); and the more negative their social interactions were in the moment, the lower was their momentary well-being (Model 5: *b* = -0.31, *p* < .001). This pattern of associations is consistent with the possibility that when feeling lonely, people tend to have more negative interactions with others and having more negative interactions is associated with lower well-being. Adding basic socio-demographics (age, gender, employment status) to the models did not alter these results. See Table S4 in the Supplementary materials.

### Discussion

Study 3 provided further support to the amplifying account, using a more recent ESM sample recruited during the pandemic: The negative association between momentary loneliness and momentary well-being was amplified in other people’s presence (vs. being alone). Interestingly, this study also showed that in moments of loneliness, being around others was associated with worse well-being than being alone.

We also found that the pattern of correlations among momentary loneliness, social withdrawal desire, interaction quality, others’ presence and experienced well-being was consistent with the two proposed mechanisms of the amplifying account. First, feelings of loneliness were associated with a stronger desire for social withdrawal, which was especially predictive of lower well-being when being in others’ company (vs. alone). Second, when feeling lonely, individuals tended to have more negative social interactions, and lower well-being.

## Studies 1–3: Lagged Analyses

Since all studies included several measurements per participant, we additionally explored whether feeling lonely while in company (vs. alone) at one time during the day affected well-being at the next assessed time during the same day. This approach can show whether the effect of loneliness in others’ presence (vs. alone) is long-lasting (i.e., extends to the subsequent hours) vs. limited to the momentary experience. We regressed well-being at *t* on loneliness at *t-1*, others’ presence at *t-1*, their interaction, and well-being at *t-1*. The interaction did not reach significance in any of the studies (see Table S5 in SOM). These results should be considered in light of the following limitation. None of the studies collected information on *consecutive* time periods. For example, in Study 1, using Day Reconstruction method, there were about 30 episodes per day per participant but only for 3 randomly selected episodes the measures of loneliness, social contact and well-being were collected. Hence, feeling lonely while with others (vs. alone) during one episode might not have affected well-being at the next assessed episode since there were many other episodes in between that could be lonely or social, that we have no data on. In combination with the robust contemporaneous effects, the absence of lagged effects might indicate that the association between loneliness and well-being in others’ presence (vs. alone) is limited to the momentary experience of well-being and dissipates quickly.

## General Discussion

Loneliness represents an increasingly common experience, with up to half of Americans reporting some degree of loneliness (HRSA, 2021). Importantly, loneliness is not only common, it is also associated with a multitude of negative outcomes for individuals’ health and well-being (Hawkley & Cacioppo, 2010). Given that loneliness is usually defined as a discrepancy between the desired and actual social relationships (Russell et al., 1980), popular science books often recommend increasing one’s social contact as a way of coping with the negative psychological consequences of feeling lonely (DCMS, 2021). But are the moments of loneliness actually more bearable when in others’ company?

The academic literature has suggested two conflicting theoretical accounts of the role of social contact in the association between loneliness and well-being. According to the buffering account, social contact could alleviate the negative impact of loneliness on well-being. In contrast, according to the amplifying account, social contact could exacerbate the negative effect of loneliness on well-being. The results of three studies (*N*_individuals_ = 3,035) using ecological momentary assessment were consistent with the amplifying account. The negative association between momentary feelings of loneliness and momentary well-being was amplified – that is, became even stronger – when people were around others (vs. alone). It is also noteworthy that when individuals felt lonely, being around others was no longer associated with higher well-being (Studies 1 and 2) or was even associated with worse well-being than being alone (Study 3).

Why wasn’t there any support for the buffering model of social interactions? We speculate that the buffering effect might be restricted to certain types of negative experiences and social interactions (Jolly et al., 2021). Specifically, most empirical support for the buffering model emerged with respect to non-social stressors (e.g., occupational stress, job demands, environmental stress) and a specific form of interactions: social support. Our findings suggest that this buffering effect does not extend to the feeling of loneliness, a social stressor, and general forms of social interactions which do not necessarily involve social support.

Why is the negative association between loneliness and well-being stronger when being around others (vs. when alone)? We proposed that moments of loneliness could be associated with a stronger desire to be alone and might therefore feel particularly aversive when one has to be around others (vs. alone). In support of this possibility, research on solitude suggests that being alone can be functional, allowing individuals to regulate their emotions and avoid undesirable interactions (Nguyen et al., 2017; Ost et al., 2021; Ren et al., 2021a, b). Unfulfilled desires for being alone is even associated with poorer wellbeing outcomes (Coplan et al., 2019a, b). In addition, when feeling lonely, people might engage in more negative social interactions which could be detrimental for well-being. Although the correlational nature of our data did not establish causality, the patterns of the relationships among the variables of interest (feelings of loneliness, the desire to withdraw, poor interaction quality and well-being) were consisted with both of these proposed explanations. Finally, there might be additional processes in place that could explain our findings as well. For example, loneliness might make it harder to establish a true sense of connection with others. Having to socialize with others without achieving a sense of connection might feel particularly draining and meaningless, damaging one’s psychological well-being. Relatedly, exploring whether there are specific types of contact (e.g., contact with someone one fully trusts) is helpful in alleviating the negative impact of feeling lonely on well-being is worth further investigation.

All three studies provided support for the amplifying model of social contact: the negative associations between momentary loneliness and momentary well-being were consistently enhanced in others’ presence (vs. absence). However, there were also differences between the studies. Specifically, although social contact (vs. alone) was associated with worse well-being outcomes at high levels of loneliness across all three studies, this negative link was only significant in Study 3. This difference was potentially due to the fact that Study 3 data were collected during a pandemic. It is possible that the typically observed positive association between social contact and well-being reverses only at extremely high levels of momentary loneliness and that such extreme levels were more likely to be observed during the pandemic (Study 3) than before (Studies 1 and 2)^4^. Additionally, the rewarding nature of social interactions may be complicated by people’s concerns about risks of infection or intentions to follow physical distancing guidelines when they came into in-person contact during a pandemic (Tybur et al., 2020; Young et al., 2021). Alternatively, the discrepancy between the studies could also be explained by different measures of social contact. While in Studies 1 and 2, we captured the mere presence of others, Study 3 measured whether participants actively interacted with others in the moment. It is not surprising that active social interactions (vs. the mere social presence) produced a stronger effect, resulting in worse well-being (compared to being alone) when feeling lonely.

While the reliance on the everyday life approach and momentary assessments increased the ecological validity of the finding, this method has restricted our ability to make causal conclusions. We observed that experiences of loneliness and poor well-being are more likely to co-occur when around others than alone. We assumed that feeling lonely leads to lower well-being, but it is also possible that poor well-being leads to more perceived loneliness. Regardless of the exact causal mechanism, the pattern of the relationships observed in the data is consistent with the amplifying account (and contradicts the buffering account) and suggests that moments of loneliness are not more bearable when spent in others’ company than when spent alone. Nevertheless, given the observational nature of the present studies, there is a need in intervention studies combining experimental manipulations of state loneliness and a random assignment to different amounts of social interactions, before any practical recommendations can be made.

Relatedly, while we have provided evidence for the proposed mechanisms underlying the amplifying effect of social contact, the precise causal relationships between momentary loneliness and momentary interaction quality await further tests that allow for strong causal inference. For example, while many studies have documented the social stigma of loneliness (Cacioppo & Hawkley, 2005), the stigma might be a result of individuals’ own negative social behavior in moments of loneliness. Consistent with this possibility, loneliness has been associated with lower trust and cooperation during small group discussions (Anderson & Martin, 1995; Rotenberg, 1994). Thus, understanding the causality behind the interrelations of loneliness, individuals’ own behavior and the behavior of their interaction partners deserves more empirical attention in the future research.

The present studies focused exclusively on everyday experiences capturing momentary states. Does the amplifying model of social interactions apply to the level of traits as well? Previous research has shown that adolescents with steadily high levels of loneliness over years (vs. adolescents who experienced loneliness only temporarily) showed a curbed emotional response to social inclusion situations (Vanhalst et al., 2015). Hence, it might be worthwhile to examine whether increasing social contact amplify or attenuate the negative association between chronic loneliness and psychological well-being.

Loneliness represents a particularly aversive experience, that is not only aversive in and of itself but also in the consequences it has on individuals’ physical and mental health. Much of the research effort has been directed at designing and testing interventions aimed at reducing the feeling of loneliness. Meta-analytic results suggest that the probably most intuitive way of fighting loneliness – seeking social company – is not the most efficient one (Masi et al., 2011). Simply bringing lonely people together might not reduce the feeling of loneliness because being social would not automatically turn lonely people into attractive interaction partners. In a similar vein, the present findings show that the negative link between loneliness and well-being was strengthened, not attenuated, when people had social companies (vs. alone).

## Electronic Supplementary Material

Below is the link to the electronic supplementary material.

Supplementary Material 1

## Footnotes

1 In all three studies, participants also indicated who was present (partner, colleagues, friends, etc.). Additional analyses did not provide consistent evidence of the effects being driven by specific categories of others (see Table S2 in SOM for details).

2 Meaning showed a poor item-scale correlation (*r* = .09); removing meaning from the scale did not affect the results (see Table S1 in SOM).

3 The questionnaire also included the same item about positive interactions but about half of the responses were lost due to a programming error.

4 We could not compare the average reported loneliness across studies due to different response scales (Studies 1 and 2: 7-point Likert scale; Study 3: 5-point Likert scale).
