# Supplementary material for: Alone in a Crowd: Is Social Contact Associated with Less Psychological Pain of Loneliness in Everyday Life?
Source: J Happiness Stud. 2023 May 4;24(5):1841–60. doi: 10.1007/s10902-023-00661-3 (PMC10157120; doi:10.1007/s10902-023-00661-3)
Supplement: Supplementary file 1 — Supplementary Material 1 [file 10902_2023_661_MOESM1_ESM.docx]

**Study 1**

**Analysis using the adjusted psychological well-being scale**

Psychological well-being scale was computed as a composite of 12 items, one of which was meaning. Because meaning showed a poor item-scale correlation (*r* = .09), we excluded meaning from the composite score and repeated the analyses we report in the main text. These additional analyses fully replicated the results reported in the manuscript (see below).

Table S1

*Multilevel regression predicting momentary psychological well-being (with “meaning” excluded from the composite score)*

|  | **Study 1** | | |
| --- | --- | --- | --- |
| *Predictors* | *b* | *95% CI* | *p* |
| Momentary loneliness | -0.18 | [-0.19, -0.17] | **<0.001** |
| Other’ presence | 0.13 | [0.11, 0.14] | **<0.001** |
| Momentary loneliness x Others’ presence | -0.04 | [-0.07, -0.02] | **<0.001** |
| N years | 4 | | |
| N individuals | 2,498 | | |
| N assessments | 21,652 | | |

*Note.* Others’ presence: 1 = with others, 0 = alone. All predictors were group-mean centered (i.e., within individuals).

**Study 3**

Attention check

Please provide an answer for each of the following questions. 
Please take the time to answer these questions seriously. Here's why, we test whether you actually take the time to read the instructions. 
Therefore, if you read this, please answer 'three' on the first question, and 'five' on the second and third question. 
These questions give us important background information about the participants.

|  | Not at all  1 (1) | 2 (2) | 3 (3) | 4 (4) | 5 (5) | 6 (6) | 7 (7) | 8 (8) | Very much  9 (9) |
| --- | --- | --- | --- | --- | --- | --- | --- | --- | --- |
| I prefer to get bonuses over steady income (1) |  |  |  |  |  |  |  |  |  |
| I think receiving a bonus within 2 weeks is fast enough (2) |  |  |  |  |  |  |  |  |  |
| I prefer experiments where I can interact with other people (3) |  |  |  |  |  |  |  |  |  |

**Effects of different social partners, Studies 1 - 3**

In all studies, participants indicated who was present during each episode: partner, colleagues, friends, clients, children, supervisor, relatives, other. Participants could select multiple categories. We created a series of dummy variables reflecting each category of other people being present (1=a specific category present, 0 = a specific category not present). We repeated the analyses presented in the main text using these dummies. The results did not provide consistent evidence of the effects being driven by specific categories of others (Table S2).

Table S2

*Effects of different social partners on momentary well-being*

|  | Study 1 | | | Study 2 | | | Study 3 | | |
| --- | --- | --- | --- | --- | --- | --- | --- | --- | --- |
|  | N (%) | *b* | *p* | N (%) | *b* | *p* | N (%) | *b* | *p* |
| Momentary loneliness x Partner’s presence | 6124 (48) | -.001 | .959 | **3793 (29)** | **-.06** | **<.001** | **3489 (44)** | **-.12** | **<.001** |
| Momentary loneliness x Friends’ presence | **897 (7)** | **-.13** | **<.001** | 1127 (9) | .001 | .939 | 1419 (18) | -.03 | .336 |
| Momentary loneliness x Colleagues’ presence | 1146 (9) | -.04 | .094 | **1504 (11)** | **.09** | **<.001** | 784 (10) | .06 | .071 |
| Momentary loneliness x Clients’ presence | 325 (3) | -.02 | .686 | 268 (2) | .04 | .448 | 328 (4) | -.04 | .363 |
| Momentary loneliness x Children’s presence | 1934 (15) | .02 | .403 | 1719 (13) | -.02 | .282 | 1450 (18) | -.01 | .795 |
| Momentary loneliness x Relatives’ presence | **745 (6)** | **-.09** | **<.006** | **709 (5)** | **-.06** | **<.025** | - | - | - |
| Momentary loneliness x Parents’ presence | - | - | - | - | - | - | 1593 (20) | -.04 | .100 |
| Momentary loneliness x Supervisor’s presence | 209 | .02 | .698 | 152 (1) | -.06 | .282 | 126 (2) | .03 | .538 |
| Momentary loneliness x “Other” category’s presence | 616 | -.05 | .096 | 831 (6) | -.05 | .047 | **544 (7)** | **-.10** | **<.009** |

*Note.* The models included the main effects of momentary loneliness and others’ presence. N (%) = number of assessments where the respective social category was present and their percentage in the overall number of assessments (including “being alone”).

**Controlling for socio-demographics, Studies 1 - 3**

Table S3

*Multilevel regression predicting momentary psychological well-being, including socio-demographics, Studies 1 and 2*

|  | **Study 1** | | | **Study 2** | | |
| --- | --- | --- | --- | --- | --- | --- |
| *Predictors* | *b* | *95% CI* | *p* | *b* | *95% CI* | *p* |
| Momentary loneliness | -0.16 | -0.17 – -0.15 | **<0.001** | -0.18 | -0.19 – -0.17 | **<0.001** |
| Others’ presence | 0.14 | 0.13 – 0.16 | **<0.001** | 0.10 | 0.08 – 0.12 | **<0.001** |
| Momentary loneliness x Others’ presence | -0.05 | -0.07 – -0.02 | **<0.001** | -0.05 | -0.08 – -0.03 | **<0.001** |
| Age | 0.003 | 0.00 – 0.00 | **<0.001** | 0.002 | -0.00 – 0.01 | 0.308 |
| Gender | 0.03 | -0.01 – 0.06 | 0.119 | -0.04 | -0.18 – 0.11 | 0.630 |
| N years | 4 | | | - | | |
| N individuals | 2,498 | | | 265 | | |
| N assessments | 21,652 | | | 12,730 | | |

*Note.* Others’ presence: 1 = with others, 0 = alone. Gender: 1 = male, 0 = female. All predictors were group-mean centered (i.e., within individuals).

Table S4

*Multilevel regression results, with socio-demographic control variables, Study 3*

|  | **Model 1** | | | **Model 2** | | | **Model 3** | | | **Model 4** | | | **Model 5** | | | |
| --- | --- | --- | --- | --- | --- | --- | --- | --- | --- | --- | --- | --- | --- | --- | --- | --- |
|  | **Momentary well-being** | | | **Momentary withdrawal desire** | | | **Momentary well-being** | | | **Momentary negative interactions** | | | **Momentary well-being** | | | |
| *Predictors* | *b* | *95% CI* | *p* | *b* | *95% CI* | *b* | *95% CI* | *p* | *b* | *95% CI* | *b* | *95% CI* | *p* | *b* | *95% CI* |  |
| **Momentary loneliness** | -0.34 | -0.36 – -0.33 | **<0.001** | 0.32 | 0.29 – 0.35 | **<0.001** | - | - | - | 0.20 | 0.18 – 0.22 | **<0.001** | - | - | - |  |
| **Others’ presence** | 0.12 | 0.09 – 0.14 | **<0.001** | - | - | - | 0.13 | 0.10 – 0.15 | **<0.001** | - | - | - | - | - | - |  |
| Age | 0.01 | 0.00 – 0.01 | **0.011** | -0.01 | -0.02 – -0.00 | **0.015** | 0.01 | 0.00 – 0.01 | **0.015** | -0.00 | -0.01 – 0.00 | 0.057 | 0.01 | 0.00 – 0.01 | **0.011** |  |
| Gender | 0.08 | -0.06 – 0.21 | 0.254 | -0.13 | -0.27 – 0.01 | 0.077 | 0.08 | -0.05 – 0.21 | 0.244 | 0.01 | -0.06 – 0.08 | 0.733 | 0.08 | -0.06 – 0.21 | 0.253 |  |
| Employment: work (company premises) | -0.01 | -0.17 – 0.15 | 0.920 | -0.02 | -0.19 – 0.15 | 0.826 | -0.00 | -0.16 – 0.15 | 0.956 | 0.02 | -0.07 – 0.10 | 0.704 | -0.01 | -0.17 – 0.15 | 0.937 |  |
| Employment: other | -0.10 | -0.32 – 0.13 | 0.391 | -0.05 | -0.28 – 0.19 | 0.701 | -0.10 | -0.33 – 0.12 | 0.353 | 0.01 | -0.11 – 0.13 | 0.872 | -0.10 | -0.32 – 0.13 | 0.393 |  |
| Employment: retired | 0.01 | -0.34 – 0.37 | 0.943 | -0.03 | -0.40 – 0.34 | 0.877 | 0.02 | -0.33 – 0.37 | 0.916 | 0.07 | -0.12 – 0.25 | 0.490 | 0.02 | -0.34 – 0.37 | 0.925 |  |
| Employment: student | 0.02 | -0.17 – 0.22 | 0.831 | -0.11 | -0.32 – 0.10 | 0.291 | 0.01 | -0.18 – 0.20 | 0.922 | -0.00 | -0.11 – 0.10 | 0.953 | 0.02 | -0.18 – 0.22 | 0.844 |  |
| Employment: unemployed | -0.28 | -0.47 – -0.09 | **0.004** | 0.07 | -0.13 – 0.27 | 0.475 | -0.28 | -0.46 – -0.09 | **0.004** | -0.07 | -0.17 – 0.03 | 0.196 | -0.28 | -0.47 – -0.09 | **0.005** |  |
| **Momentary loneliness x Others’ presence** | -0.11 | -0.16 – -0.07 | **<0.001** | - | - | - | - | - | - | - | - | - | - | - | - |  |
| **Momentary withdrawal desire** | - | - | - | - | - | - | -0.26 | -0.28 – -0.25 | **<0.001** | - | - | - | - | - | - |  |
| **Momentary withdrawal desire x Others’ presence** | - | - | - | - | - | - | -0.19 | -0.23 – -0.15 | **<0.001** | - | - | - | - | - | - |  |
| **Momentary negative interactions** | - | - | - | - | - | - | - | - | - | - | - | - | -0.30 | -0.32 – -0.28 | **<0.001** |  |
| N individuals | 265 | | | 265 | | | 265 | | | 265 | | | 265 | | | |
| N assessments | 7711 | | | 7361 | | | 7360 | | | 7715 | | | 7710 | | | |

*Note.* Others’ presence: 1 = with others, 0 = alone. Gender: 1 = male, 0 = female. For employment, work (home office) is the reference category. All predictors were group-mean centered (i.e., within individuals).

Table S5

*Multilevel regression predicting momentary psychological well-being using lagged values of loneliness and others’ presence*

|  | **Study 1** | | | **Study 2** | | | **Study 3** | | | |
| --- | --- | --- | --- | --- | --- | --- | --- | --- | --- | --- |
| *Predictors* | *b* | *95% CI* | *p* | *b* | *95% CI* | *p* | *b* | *95% CI* | *p* |  |
| Lagged loneliness | 0.09 | 0.07 – 0.12 | **<0.001** | -0.01 | -0.02 – 0.01 | 0.298 | 0.14 | 0.11 – 0.16 | **<0.001** |  |
| Lagged others’ presence | -0.15 | -0.18 – -0.12 | **<0.001** | -0.01 | -0.03 – 0.01 | 0.371 | -0.04 | -0.08 – -0.01 | **0.010** |  |
| Lagged psychological well-being | 0.60 | 0.59 – 0.62 | **<0.001** | 0.43 | 0.42 – 0.45 | **<0.001** | 0.53 | 0.50 – 0.55 | **<0.001** |  |
| Lagged loneliness x lagged others’ presence | 0.04 | -0.01 – 0.10 | 0.115 | 0.01 | -0.02 – 0.03 | 0.592 | 0.07 | -0.01 – 0.15 | 0.073 |  |
| N years | 4 | | | - | | | - | | | |
| N individuals | 2,485 | | | 265 | | | 268 | | | |
| N assessments | 14,276 | | | 12,465 | | | 6,112 | | | |

*Note.* Others’ presence: 1 = with others, 0 = alone. All predictors were group-mean centered (i.e., within individuals), except for lagged psychological well-being (as group-mean centering is not recommended for autoregressive effects; Hamaker & Grasman, 2015). Note that the lagged effect of loneliness is positive (higher loneliness at t-1 is associated with more well-being at t) and the lagged effect of others’ presence is negative (being around others at t-1 is associated with less well-being at t). Since these predictors were centered within-persons, we speculate that this could be a result of the “regression to the mean” effect (see Stavrova et al. 2021 for a similar artefact): the higher a person’s loneliness at t-1, the lower their loneliness at t; this – together with the fact that loneliness at t is associated with less well-being at t – likely explains why loneliness at t-1 is associated with more well-being at t. Note that using noncentered values result in a negative effect of loneliness and a positive effect of others’ presence - consistent with the results presented in the main manuscript.

References

Hamaker, E. L., & Grasman, R. P. P. P. (2015). To center or not to center? Investigating inertia with a multilevel autoregressive model. *Frontiers in Psychology*, *5*. https://doi.org/10.3389/fpsyg.2014.01492

Selig, J. P., & Preacher, K. J. (2008). *Monte Carlo method for assessing mediation: An interactive tool for creating confidence intervals for indirect effects [Computer software]. Available from http://quantpsy.org/.* In

Stavrova, O., Ren, D., & Pronk, T. (2021). Low Self-Control: A Hidden Cause of Loneliness? *Personality and Social Psychology Bulletin*, 01461672211007228. https://doi.org/10.1177/01461672211007228
